# Dental care staff’s experience with risk assessment of dental erosion: a qualitative study

**DOI:** 10.1186/s12903-024-04700-0

**Published:** 2024-08-11

**Authors:** Johannes Todorov, Elena Shmarina, Annsofi Johannsen

**Affiliations:** 1 Division of Oral Health and Periodontology, Department of Dental Medicine, Karolinska Institutet, Stockholm, Sweden; 2 Kronoberg County Council, Public Dental Service, Växjö, Sweden; 3 Kalmar County Council, Public Dental Service, Oskarshamn, Sweden

**Keywords:** Tooth wear, Health promotion, Dental prophylaxis, Oral health, Dental ethics

## Abstract

**Background:**

The risk assessment of dental erosion among children and adolescents is an important aspect of dental care, as dental erosion constitutes a rapidly growing, global problem. Dental professionals rely solely on their own perception, as the current risk assessment process is not completely automatized, which affects the risk assessment reliability.

**Aim:**

To explore dental professionals’ experiences with risk assessment of dental erosion among children and adolescents.

**Method:**

In-depth interview was used as data collection method. A total of 11 dental professionals were interviewed. The interviews were analyzed using qualitative content analysis.

**Results:**

The findings were summarized in the categories *Professionals’ responsibility*,* Systematic approach *, and *Collaboration and communication*. Dental staff perceived that their basic knowledge regarding erosion should be improved, and skills development was desired to reduce the knowledge gaps around the risk assessment of dental erosion. They alleged that the systematic approach could be improved by reducing workplace stress, implementing a universal dental erosion index, improving the existing risk assessment software, and automating the risk assessment of the condition. Dental professionals also experienced a need to calibrate and collaborate with each other and with other healthcare professionals to improve patient care.

**Conclusion:**

Dental professionals experienced their basic knowledge of dental erosion and their risk assessment as good, but a more advanced skill development was required. Furthermore, they experienced the risk assessment software as a good tool that should be improved to compile more objective risk assessment. A universal erosion index was also requested.

## Background

Dental erosion is one of the most common causes of tooth wear and constitutes a rapidly growing, global problem [1]. Erosion occurs as a result of a non-bacterial chemical action of acid on the tooth surface [2] and appear initially in the enamel. At a later stage, the damage can spread to the dentin and lead to irreversible tooth substance loss [3–5]. The loss of tooth substance leads to functional problems which can have serious consequences for children and adolescents. Dental erosion can often cause pain, which may negatively affect the patient’s quality of life [5]. Sensitive teeth and chewing defects caused by dental erosion can affect patient’s eating and drinking ability, and cause malnutrition [1]. This, in turn, place additional burdens on families and challenge healthcare teams dealing with managing these negative effects. The severe loss of tooth substance in the case of extensive dental erosion also requires extensive prosthetic treatment [6]. This treatment is provided at no cost for patients in Sweden as dental care is free of charge for individuals up to the age of 23 [7]. The substantial expenses associated with prosthetic treatments, however, are funded by public dental health services, and thus imposes a significant financial burden on the Swedish welfare system. While in Sweden, this may constitute a problem for the society, in other countries without similar reimbursement system, it could lead to extensive economic strains for individuals. Therefore, it is imperative to reduce the prevalence of dental erosion to alleviate this economic strain.

The global increase in soft drink consumption, changed lifestyles, and increased incidence of chronic diseases are some of the reasons for higher incidence, prevalence, and severity of dental erosion among children and adolescents in recent years [1, 8, 9]. Studies from Europe, India, Cuba, Sudan, and Saudi Arabia report prevalence of dental erosion among children and adolescents between 2 and 53%, while in Sweden the numbers vary between 12–22% [1]. The prevalence of dental erosion that spreads into the dentin is 8–34% among children between 2 and 7 years of age, and 2–53% in adolescents between 11 and 20 years, explained by increasing age [1].

Dental erosion is one of several oral diseases and conditions that is risk-assessed in Sweden to increase patient safety [7]. Various risk assessment software, such as Caries Management by Risk Assessment (CAMBRA), Cariogram, and Decision Support R2, are widely used in dental care centers in Sweden [10–12]. However, only R2, which is a Swedish risk assessment software measures the risk of dental erosion. R2’s main disadvantage is that it lacks functions to import information about certain diagnoses, including dental erosion, or risk factors for dental erosion, such as diet, medications, diseases etc., from the dental record software. As a result, the dental erosion risk assessment is not carried out by the risk assessment software but depends entirely on the dental professional’s knowledge, interpretation of the risk factors, attention, and previous experience, which usually somewhat vary. The variation of different indices and scales that are used for registering dental erosion makes the risk assessment of dental erosion even more difficult [13, 14]. This leads to a large variation of dental erosion risk assessment with unclear quality. The differences in the risk assessments affect the choice of health promotion, dental prophylaxis, and treatment, which affects dental erosion prevalence and spread.

Despite the knowledge about dental erosions, highly trained personnel and access to risk assessment software, the prevalence of erosions continues to rise, negatively affecting both children and their families and placing a strain on the Swedish welfare system. A correct risk assessment, conducted in a timely manner, is important to prevent dental erosion occurrence and prevalence. To ensure relevant and high-quality preventive measures for children and adolescents with dental erosion, and those at risk of dental erosion, the experiences of licensed dental professionals should be compiled and used for improvement of existing risk assessment tools. The synthesis of diverse insights, knowledge and experiences among dental professionals can present a promising opportunity for enhancing current risk assessment software. Furthermore, such collaborative efforts have the potential to refine existing guidelines for assessing dental erosion, thereby indirectly alleviating its adverse consequences. Qualitative research can facilitate an in-depth exploration of dental professionals’ experiences and perceptions, providing valuable insights that can generate new outcomes. Hence, it was chosen as the methodological approach for this study. To our knowledge, no study to date has investigated this matter. The problems with erosion are highly contemporary and require extra efforts, as noticed by Sweden’s Regions in the National System for Knowledge Management Health Care at the beginning of 2023 [15].

### Aim

To explore dental care professionals’ experience with risk assessment of dental erosion among children and adolescents.

## Methods

The present study is a qualitative in-depth interview study, analyzed with inductive content analysis defined by Graneheim and Lundman [16]. A Consolidated Criteria for Reporting Qualitative Research (COREQ) was used as guideline [17]. The researchers emphasized that information saturation depends on data relevance to the study’s aim, rather than the numbers of participants involved [18]. Consequently, the sample size was established based on this criterion. New participants were interviewed until theoretical saturation of information was reached, ensuring that no additional insights or variations in the data emerged [19].

### Settings

The study was conducted at the Public Dental Health Service (PDHS) in Kalmar County, Sweden. Approximately 400 dental care staff take care of nearly 247,000 inhabitants in the county’s three specialist clinics and 18 general public dental clinics.

### Participants

Purposive sampling was used in the present study. Purposive sampling was chosen as it provides greater opportunities to select participants with superior experience of risk assessment of dental erosion, which gives the opportunity to collect highly substantial information that can correspond to the aim of the study [20]. The sample consisted of registered dental professionals employed at PDHS in Kalmar County. All participants met the following inclusion criteria: dental professional staff registered at Swedish Social Welfare Board with at least one year of work experience with risk assessment software R2 and at least one year of work experience with children and adolescent examinations. The recruitment of participants took place between April and August 2023. All the participants were recruited by assistance of PDHS managers and personal contacts. An initial email invitation was sent to all the registered dental care professionals employed at general public dental care clinics in Kalmar County. All the interested participants were contacted by the first author to confirm that they met the inclusion criteria.

A total of 11 registered dental care professionals, three dentists and eight dental hygienists from general public dental care clinics in Kalmar County participated in the present study. In Sweden both professions are qualified to diagnose erosions and are encouraged to risk assessment for dental erosion [7]. However, both professions have different roles and perspectives; hygienists often focus on preventive care and maintenance, while dentists handle more complex diagnoses and treatments [21, 22]. Two participants were men and nine were women. Participants’ working experience ranged between one and 30 years. Nine of them had received their degree in Sweden and two had received their degree in other European countries.

### Data collection

Data collection was conducted with individual in-depth interviews by the first author. An interview guide presented in Table 1 was initially designed by the first author and thereafter developed in consultation with ES and AJ. All the questions were open-ended, based on the aim of the study, and addressed dental professionals’ experiences with risk assessment of dental erosion.

The participants were also prompted to share their experience regarding the efficacy and reliability of R2. They were even encouraged to give a suggestion that might improve the existing risk assessment process. Clarifying and supplementary questions as “Could you please tell me a bit more?” and “How did it feel?” were asked when the answers were considered unclear or incomplete, or when new insights and additional information arose.

**Table 1:** Interview guide

•How do you perceive your role when it comes to risk assessment of dental erosion? •What is your opinion regarding the importance of dental erosion risk assessment and why? •Do you perform dental erosion risk assessment on all children? If not, why? If yes, how do you proceed?
•How do you assess your knowledge regarding dental erosion and its assessment? If insufficient, how can you improve it? •To what extent do you use the established risk assessment of dental erosion in your profession?

The interviews were conducted at the participants’ workplace, during their working hours in an undisturbed room of their choice, to ensure confidentiality [23]. Seven interviews were conducted using the video conference platforms Zoom or Skype, while four participants requested face-to-face interview. All the interviews continued until saturation was reached [24], and ended with a closing question that gave the participants the opportunity to add any additional information that had not been covered during the interview [23, 25]. Recruitment and interviewing were gradually increased until information saturation was reached, and additional interviews did not add anything new that affected the results [19, 26]. The interviews lasted from 54 to 80 min (median 60 min). Each interview was transcribed by the first author shortly after the recording to ensure accuracy in interpreting the information.

### Data analysis

The information collected by the interviews was compiled and analyzed using qualitative content analysis with an inductive approach to find similarities and differences in the participants’ experience of the phenomenon, as described by Graneheim and Lundman [16]. Each interview was transcribed to obtain a better basis for understanding the participant’s experience regarding the risk assessment of dental erosion. All transcriptions were read verbatim, and the material was reduced by sifting out the most essential content from the irrelevant information. The relevant information was organized into meaning units, which consisted of sentences that responded to the aim. Furthermore, all meaning units were condensed to condensed meaning units. The manifest content of the condensed meaning units was abstracted and described with codes. The codes with similar content were stratified into subcategories that were ultimately summarized to categories that composed the manifest content of all the interviews. The categories were then evaluated and analyzed to formulate into a theme, which is the study’s underlying main meaning or the latent content at an interpretive level [16]. The entire analysis process was initially conducted by the first author and subsequently discussed by all authors (ES and AJ have extensive experience in qualitative methodology) until a final consensus was reached.

## Results

The complied results regarding dental care staff’s experiences with risk assessment of dental erosion among children and adolescents formed to three categories with seven associated subcategories: *Professionals’ responsibility*,* Systematic approach*,* and Collaboration & communication.*

The underlying message that summarizes the essence of all interview data formed the theme of the study: the staff appreciates their knowledge and software, but desires both skills development and existing software improvement. The theme described the participants’ views of a self-perceived certain level of knowledge regarding risk assessments of dental erosion, in contrast to their willingness to engage in further skill development and get an improved risk assessment software that will increase the objectivity of dental erosion risk assessment and improve dental care overall. The theme and the summarized categories, along with their corresponding subcategories, are presented in Table 2. To support the analysis and illustrate the data interpretation, direct quotations from the participants are included. All interviews were identified with the abbreviation D for dentist and DH for dental hygienist, and randomly selected numbers were ascribed to distinguish them and to guarantee participants’ confidentiality. The same identification is used for the direct quotations in the results.

**Table 2:** Overview subcategories, categories, and theme

Subcategories	Categories	Themes
Awareness of the problem	Professionals’ responsibility	The staff appreciates their knowledge and software, but desires both skills development and an existing software improvement.
Perceived need for skills development		
Tools to follow up/improve	Systematic approach	
Stress and time constraints		
Sidelined parameter		
Collegial consensus	Collaboration and communication	
Interprofessional collaboration		

### Professionals’ responsibility

The category “Professionals’ responsibility” highlights dental professionals’ perception of their own responsibility, experience, skills, and knowledge, which are crucial for conducting a dental erosion risk assessment. Participants emphasized the importance of knowledge, personal responsibility, and awareness of the problem as a key factor for conducting an objective dental erosion risk assessment, which can increase patient safety. The category “Professionals’ responsibility” includes two subcategories: *Awareness of the problem* and *Perceived need for skills development*.

#### Awareness of the problem

The participants considered knowledge about dental erosion, and awareness of the problem, as important concepts related to professionals’ responsibility. They emphasized that the quality of risk assessments depends on the therapist’s knowledge of dental erosion’s etiology, risk factors, and awareness of their severity. All participants pointed out that a correct medical history and diet information are of great importance for risk assessment of dental erosion, and also important is to ask as many questions as possible during the examination, to gain a better understanding of patient’s habits, medical condition, diet etc. “You connect to their habits and then they often say that they drink a lot of soft drinks or energy drinks and then you see the erosions” (DH5).

Some participants mentioned the relation between specific diseases and dental erosion, which indicated an even deeper knowledge regarding dental erosion. “Certainly, the most important aspect for the patient is information, such as dietary habits and so forth. For some, this can indeed be a problem. Additionally, the severity of erosion damage may be influenced by factors such as potential eating disorders affecting the patient” (DH7).

Health promotion and dental prophylaxis were mentioned as important factors to prevent dental erosion and that were also connected to risk assessment and its purpose. The participants perceived that many patients are often unaware about their own dental erosion, and informing them about the condition while they carrying out the risk assessment was of great importance and an essential part of professionals’ responsibility. *“*Very important to inform, because many young people consume a lot of corrosive drinks, and they don’t know about it” (DH1). “They consume a lot of soda or energy drinks on a daily basis” (DH5).

The participants emphasized their significant role in dental erosion risk assessment, as only a dental care professional can diagnose the condition. The participants conducted dental erosion risk assessment during the examination; however, they were doubtful whether their colleagues had the same approach when it comes to dental erosion, indicating a concern about variability in practice and the potential neglect of patient care. “It’s important that it’s included, that you assess it. I don’t think everyone takes into account that and even fill in that part of the risk assessment” (DH8).

Some participants perceived that erosion was not a major part of their responsibility and, even though they carried out the risk assessment in general, they did not pay much attention to the dental erosion risk itself. “Even if I assess the risk of dental erosion, I do not discuss the erosions with my patients.” (D9).

#### Perceived need for skills development

All participants perceived themselves as having a good basic knowledge of erosion etiology and the risk assessment thereof. When prompted to self-assess their knowledge, they rated it as average. However, updating and improvement of existing knowledge gaps as part of professionals’ responsibility was desired. Some participants described their knowledge of dental erosion as insufficient to compile an objective risk assessment and even experienced difficulties distinguishing different types of tooth wear, which affects patients’ safety, thus competence and skills development were required. “It can look like erosions, but it can also be attrition, and it can sometimes be difficult to determine if it is both parts” (DH5). “And sometimes it can be difficult to determine if it’s cuppings or something else. Some people find it hard to distinguish, like if the enamel loss is due to teeth grinding or something else” (DH8).

As new products that cause dental erosion are emerging rapidly, an update about new possible causes of erosion was considered much needed by the participants to ensure the performance of a qualitative and objective risk assessment. Some participants experienced lack of knowledge as dental erosion understanding was insufficient when they graduated, and the programs were not as focused on erosion as they are today. “I took a course 10 years ago, it was possibly just a full-day course organized by the Dental Association… But that was a long time ago, and I think these are difficult questions.” (DH4). This led to an uncertainty and insecurity that could affect the risk assessment because the therapists, due to ignorance, chose to ignore erosion and not risk assess the condition carefully. “Maybe I’m bad with these routines because I lack the habit of recording erosion … It has been so long since I went to school, and there was so little information about dental erosion” (D9).

Several participants highlighted the importance of using the risk assessment in practice to reduce the prevalence and incidence of dental erosion. Most of them informed patients about the risk factors and performed preventive measures. Others expressed that the risk assessment of dental erosion lacks a practical function, as they perceived their own knowledge of possible treatments of dental erosion and intervals for health promotion and dental prophylaxis as being uncertain and limited. “More knowledge about what to do afterwards. How often can I make an appointment with the patient … information on how to proceed after the risk assessment is compiled” (DH8). Participants emphasized the importance of not only conducting comprehensive risk assessments of dental erosion but also possessing the knowledge and ability to effectively implement treatment based on the assessment findings.

Some participants experienced insecurity about carrying out the dental erosion risk assessment. Despite the knowledge about dental erosion, the etiology, risk, and protective factors, the participants experienced difficulties in filling in the information in the risk assessment software, which affected the quality and objectivity of the risk assessment. They highlighted the gap between theoretical knowledge and practical application in dental erosion risk assessment, exacerbated by challenges with software utilization. “You are stuck. You know what dental erosion looks like, but you don’t have knowledge on how to convert the diagnosis to R2, how to register it in R2” (D11).

Participants found the scoring system used in the risk assessment of dental erosion to be confusing, which in turn undermines the risk assessments’ reliability. While the assessment of caries benefits from a structured scoring system, erosion assessment presents challenges due to its variable nature. They made a comparison between caries and erosion, where the caries risk changes depending on the number of cavities and spread, while with erosion it is unclear how the dental care professional can adjust the risk based on the number of eroded surfaces. “With caries, the more surfaces you have, or the deeper they are, you collect points. But not with erosion … it’s a bit difficult” (DH4).

### Systematic approach

The systematic approach is an important part of the working process that facilitates the workflow and is an underlying factor for conducting a qualitative dental erosion risk assessment, according to the participants. They highlighted several important aspects of the systematic approach that should be improved to facilitate the working process. The category “Systematic approach” consist of three subcategories: *Sidelined parameter*,* Stress and time constraints*, and *Tools to follow up/improve.*

#### Sidelined parameter

Several participants perceived dental erosion as sidelined compared to other diagnoses. Dental erosion was often considered to be an overlooked parameter when it comes to risk assessment and patient treatment in the form of health promotion and dental prophylaxis. A care program for dental erosion among children and adolescents is missing, which causes confusion as to whether the condition should even be risk assessed and treated or not.

The participants raised the benefits of dental erosion risk assessment and shared the same opinion that dental erosion risk assessment is positive and worthwhile. However, they perceived that the caries risk usually takes precedence when it comes to risk assessment, health promotion, dental prophylaxis, and treatment. “I believe that the caries risk is considered more than the risk of erosion” (DH6). “Caries has been around for a long time, we have knowledge, we have research, and education, and we know how it develops. But dental erosion… is different” (D9).

Several participants said that during their education they learned about prosthetic and non-prosthetic treatment, and dental prophylaxis for patients with dental erosion. However, they experienced a level of uncertainty as dental erosion is not included in children and adolescent care programs, which undoubtedly affects the systematic approach. “With caries we have a care program that must be followed, with erosion we have nothing …You do what you want. There are no guidelines telling you what to do. Some colleagues do prophylaxis, some do nothing” (DH10).

#### Stress and time constraints

Some participants perceived that stress and time constraints that occur at work affect the systematic approach and undermines the risk assessments’ reliability, which in turn affects patients’ safety. They said that in stressful situations the risk assessment of dental erosion can be absent, which emphasized the importance of a less stressful work environment. “It can go wrong, especially if you are stressed, then you might forget to do the risk assessment” (DH1). “We have so many other tasks; examinations, treatments, informing the patients…” (D3).

Other participants experienced that time constraints are a main reason not to adjust the dental erosion risk assessment. That affects risk assessments objectivity as dental erosion risk assessment is not conducted automatically and requires a manual adjustment. “It goes way too fast. Because of time constraints, you may forget to adjust the dental erosion manually in R2 before compiling the risk assessment” (D3). Because of rushed workflow, crucial steps in the assessment process may be overlooked or not given sufficient attention. This oversight could potentially lead to incomplete or inaccurate risk assessments, highlighting the need for streamlined workflows and efficient time management strategies in clinical practice. “It is a lot, and it takes time of course” (DH7).

#### Tools to follow up/improve

The participants’ overall perception of dental erosion risk assessment compiled with R2 was that it is an important part of the overall risk assessment, and it can benefit the patient and PDHS’s finances. The participants considered R2’s functionality as low and required a certain software improvement. The participants perceived that a guideline with clear language and consistent terms for dental erosion assessment, automation of R2, and synchronizing with the dental record software could improve the systematic approach and improve dental erosion risk assessment’s objectivity.

The risk assessment software R2 was described as sliding and blunt, while the risk assessment of dental erosion was perceived as unclear. The majority expressed dissatisfaction as R2 retrieves information regarding some diagnoses and conditions from the dental record software and compiles the risk assessment automatically, but dental erosion was not one of them. “R2 should retrieve the information we enter in the dental record software, about diagnosis, which surfaces, which teeth, and which grade. It should follow automatically, as it does with caries” (D3). “I wish that it was like with caries” (DH4).

Several participants noticed that R2 does not take acidic diet into account, which affects the risk assessment reliability. They requested that diet as a modifying factor in R2 would also affect the erosion risk and not do so just caries, as it does at present.

Both R2 and the dental record software use different terms for diagnosing and grading dental erosion. That makes the risk assessment of erosion even more difficult and can compromise the risk assessment, according to the participants. *“*We use one scale when we register erosions in the journal, which doesn’t quite match R2’s scale. R2’s lowest grade for erosion says no erosions, while the lowest grade in the dental record software is none or mild erosion … not the same terms used …” (DH4). “It is a bit confusing. It is not perfectly aligned, in my opinion” (DH4).

Some of the participants expressed that they also lack guidelines for examination of dental erosion, while others were aware that those guidelines exist. It was just difficult to find them as they were fragmented and not compiled in the same place on the employer’s intranet, which complicates the risk assessment conduction. “It’s not even on the intranet, it’s on the Kalmar County healthcare website … I don’t think anyone has ever mentioned that we have these guidelines there” (DH4). “We have nothing to rely on when it comes to erosions” (DH10).

### Collaboration and communication

Collaboration and communication were something that almost all participants perceived as important, and it was highlighted several times during the interviews. The patient’s safety is of great importance to the participants, and to ensure safety, collaboration and communication between dental professionals was considered desirable.

The category “Collaboration and communication” combines the two subcategories: *Collegial consensus* and *Interprofessional collaboration*.

#### Collegial consensus

Some participants emphasized that consensus between dental practitioners can increase the objectivity and quality of dental erosion risk assessment. They perceived that all of them work differently and considered a calibration between colleagues as important. The participants believed that calibration could give them a sense of security, knowing that they think in the same way as other coworkers. “I often meet a patient who’s had a risk assessment done by another dental practician. And the assessment does not match mine” (DH4). “A little more calibration, it’s also important that we work in a similar manner as much as possible” (D3).

#### Interprofessional collaboration

Some participants suggested that collaboration between colleagues could contribute to an exchange of skills that could generate profits for both patients and PDHS. Dental hygienists perceived that the collaboration with the dentists is absent sometimes and they wished for an improvement in communication and deeper collaboration with them. “Support from dentists can be a little lacking” (DH8).

Several participants perceived that patients are not always honest about their diet, which can affect the risk assessment’s objectivity. Collaboration with other professions within healthcare could contribute to widespread access to detailed information regarding dental erosion and improve the risk assessment’s objectivity. Interprofessional work with speech therapists was considered as an appropriate measure. “It would be good if it wasn’t just the dental professionals that talked about erosion. We could get support from the health care, for example a speech therapist or others who see this as a problem in the mouth” (DH4).

## Discussion

This study explored dental care professionals’ experiences with risk assessment of dental erosion among children and adolescents. Participants’ overall perception was that risk assessment of dental erosion is an important part of their professional role to prevent oral diseases and enable patients to achieve better outcomes in the form of better lifestyle and health.

Participants expressed good prerequisites and a great basic knowledge to carry out risk assessments of dental erosion on children and adolescents, rating their knowledge as average. However, they also reported some challenges that affect the risk assessment’s reliability. All the challenges that emerged during the interviews have a great potential for improvement and include aspects such as skills development, attitudes towards dental erosion, improvement of existing tools for risk assessment, collaboration and cooperation, and reducing both work-related stress and time constraints.

Furthermore, participants experienced a high degree of awareness regarding dental erosion along with good basic knowledge to detect, register, and risk-assess erosion, and to inform patients, which is in line with their competency description [21, 22] and is also supported by other studies [27, 28]. However, previous studies [27, 28] focus solely on the experiences of dentists and do not comprehensively cover the entire dental professional team involved in managing dental erosion. The present study provides a broader perspective by incorporating insights from both dentists and dental hygienists, thus contributing to new viewpoints on the experiences and knowledge of dental hygienists in detecting, recording, and informing patients about dental erosion. Additionally, the participants in this present study reported possessing a certain level of knowledge for risk assessing the condition, a factor that has not been addressed in previous studies [27, 28].

An increased knowledge to improve professional competence regarding erosion and their risk assessment was important, according to the participants. Some participants experienced difficulties diagnosing dental erosion as in the past it was not emphasized in detail in their educational programs, as it is today, which was also stated by other authors [29, 30], and can affect the risk assessment’s reliability. This educational gap can potentially impact the reliability of risk assessments. The feeling of inadequacy because of a perceived lack of competence can lead to lowered work motivation, which can affect the professional’s work and lower the risk assessment’s reliability, which was also acknowledged by Kristoffersen [31]. Therefore, continuous skills development is very important to ensure high-quality risk assessment.

The purpose of risk assessment is to identify high-risk patients and offer them appropriate care. Some participants experienced, however, competence deficiency about treatment options as well as dental prophylaxis as there is no standard procedure that can be recommended today, which also was stated by Mulic et al. [27]. Previous research [27] focused exclusively on the perspectives of dentists and indicated that little priority was given to documentation and dietary analyses. In contrast, both dental hygienists and dentists in the present study prioritized these aspects, considering them crucial for further screening and monitoring of dental erosion, which was recommended by Milosevic [32].

National guidelines for dental care have been drawn up by the National Board of Health and Welfare to be implemented in regional and local care programs, to support and guide dental care staff regarding various decisions concerning dental prophylaxis and prosthetic treatments i.a. [7]. Not including dental erosion in the care program for children and adolescents [33] implied that erosion is a neglected parameter and not as prioritized as caries and other diagnoses, as also mentioned by Curtis et al. [29]. This raises doubts about whether the diagnosis should be noticed and treated or not, and disrupts the systemic approach because the care efforts must be designed according to the care programs [7].

Work-related stress among dentists can lead to incorrect decision-making and treatment, which has also been confirmed in a study by Yansane et al. [34]. The present study, however, included both dentists and dental hygienists, and the findings demonstrate that stress adversely affects decision-making in both groups. Dental erosion risk assessment was not always conducted because of work-related stress and time constraints, according to the participants. Due to that, children and adolescents with dental erosion or risk for erosion did not receive an appropriate health assessment, dental prophylaxis, or treatment; this lack may have affected their lifestyle and entailed a higher cost for prosthetic treatments that could have been avoided if the risk assessment had been compiled [35]. A systematic risk assessment should be prioritized as it can facilitate prevention and lead to improved oral health, according to the National Board of Health and Welfare [7] thereby reducing the risk of potentially extensive and costly prosthetic treatments [36] .

The risk assessment of dental erosion carried out with R2 is not automated, thus the software does not fulfill its function. For dental professionals to be able to trust the risk assessment software and use it more frequently and effectively, the tool needs to be improved according to the wishes of the users. The participants requested that diet, as a modifying factor in R2, should affect the risk assessment of dental erosion and that R2 should retrieve information about the erosion and its distribution from the dental record software and compile the risk assessment automatically, as it does with the risk assessment of other diagnoses. The participants mentioned that one and the same prevalence of dental erosion is referred to by different terms in the dental record software and R2. This affects the validity of the diagnosis, complicates the risk assessment process, and leads to an ambiguity that can generate incorrect risk assessments, which was also mentioned in a study by Ganns [37]. Using the same terms for one and the same condition and diagnosis is something that will reduce the ambiguity surrounding the registration of dental erosion and improve risk assessment.

The validity of diagnostic tools used to identify dental erosion before compiling the risk assessment might also affect the assessments’ reliability. Eccles index, Lussi index and Basic Erosive Wear Examination (BEWE) are indices used worldwide for diagnosing dental erosion [13, 14]. However, each of these indices has demonstrated certain limitations, and none can be considered a universal standard for diagnosing dental erosion [14]. Although this issue was not raised by the participants in this study under consideration, it is a question that should be addressed in future research.

The participants perceived that many of them worked in different ways, and risk assessments of dental erosion performed by different practitioners often showed differences and low agreement. The outcome of different risk assessments can lead to uncertainty among the participants and can affect the process. In order to improve the risk assessment, a calibration of the dental staff was considered necessary, which was also mentioned by Young et al., [38] in a study covering risk assessments of caries. The present study, however, reveals that calibration is equally important when assessing the risk of dental erosion.

The importance of interprofessional collaboration that benefits many professions in dental, health, and medical care was mentioned by several participants and has been discussed in several studies [39–41]. Dental hygienists wanted more support from the dentist while carrying out the risk assessment of dental erosion to feel more certain about their decisions, while dentists expressed that help from other professions in the healthcare system, for example speech therapists, could contribute to an improved agreement around dental erosion risk assessment.

### Limitations

Combining remote and in-person interviews is a protentional limitation in this current study. Remote interviews may lack the non-verbal cues and contextual richness of in-person interviews, while in-person interviews may be influenced by the physical presence of the interviewer [42]. To address this limitation and minimize potential interpretative biases, we conducted a thorough content analysis that accounted for the varying forms of interaction and subjectivities encountered in the methodological approach. All interviews, whether remote or in-person, were transcribed verbatim and coded using the same coding framework to ensure consistency in how data were analyzed and interpreted. The results describe variations in the experiences of participants in a heterogeneous group in terms of gender, age, and experience, which strengthens the validity of the study, according to Graneheim & Lundman [43]. The results could be further affected if the inclusion criteria had also included private dental clinics and specialist clinics. The sample in the study was expanded step by step until information saturation was reached and additional interviews did not add anything new that affected the results, which is also recommended by Denscombe and Saunders et al. [19, 26]. All the interviews followed the interview guide and were transcribed the same day or promptly after the recording by the first author, to avoid overlooking valuable information and to ensure accuracy when interpreting the information [44]. To ensure the validity of the analysis, and reliability and credibility of the study, the first author discussed the stance regarding meaning units, codes, subcategory and category designations, levels of abstraction, and data saturation with the other two authors until consensus was reached, which is considered an additional strength of the study [43]. The findings are based on 11 participants but it doesn’t necessarily decrease the study’s transferability since in qualitative research the number of participants is not the decisive aspect that governs transferability [45].

## Conclusions

These findings showed that dental professionals perceived dental erosion risk assessment as an essential task. The staff rated their knowledge of dental erosion and its risk assessment as average, but more advanced skills development and staff calibration are required to facilitate the risk assessment process and increase its reliability. Furthermore, they experienced the risk assessment software as an important tool that should be improved and automatized to compile more objective risk assessment. A universal erosion index was also demanded. Further research is needed on dental professionals’ experiences in private dentistry to capture additional improvement opportunities.

## Data Availability

All datasets used and analyzed during the current study are available from the corresponding author on reasonable request.

## Abbreviations

CAMBRA: Caries management by risk assessment
PDHS: Public Dental Health Service
R2: Decision support software
